# Reproducible Boolean model analyses and simulations with the CoLoMoTo software suite: a tutorial

**DOI:** 10.1098/rsfs.2025.0002

**Published:** 2025-08-22

**Authors:** Vincent Noël, Aurélien Naldi, Laurence Calzone, Loic Paulevé, Denis Thieffry

**Affiliations:** ^1^ Institut Curie, Université PSL, 75005 Paris, France; ^2^ INSERM U1331, 75005 Paris, France; ^3^ Mines ParisTech, Université PSL, 75005 Paris, France; ^4^ Département de Biologie, Ecole Normale Supérieure, PSL Université, 75005 Paris, France; ^5^ LaBRI, CNRS UMR 5800, INP, Université de Bordeaux, 33522 Talence, France

**Keywords:** reproducible, Boolean, models, simulations, *CoLoMoTo*, tutorial

## Abstract

This tutorial provides stepwise instructions to install over 20 tools, written in multiple languages. Their integration in the *CoLoMoTo* software suite makes them accessible with a single popular language (*Python*), thereby enabling reproducible and sophisticated dynamical analyses of logical models of complex cellular networks. The tutorial specifically focuses on the analysis of a previously published model of the regulatory network controlling mammalian cell proliferation. It includes chunks of *Python* code to reproduce several of the results and figures published in the original article, and further extends these results with the help of selected tools included in the *CoLoMoTo* suite. The tutorial covers the visualization of the network with the tool *GINsim*, an attractor analysis with *bioLQM*, the computation of synchronous attractors with *BNS*, the extraction of modules from the full model, stochastic simulations of the wild-type model and of selected perturbations with *MaBoSS* and finally the delineation of compressed probabilistic state transition graphs. The integration of all these analyses in an executable *Jupyter Notebook* greatly eases their reproducibility, as well as the inclusion of further extensions. The notebook provided along with this tutorial further constitutes a template, which can be enriched with other *ColoMoTo* tools, to develop comprehensive dynamical analyses of various biological network models.

## 1. Introduction

Since the seminal studies from Sugita [1], Kauffman [2] and Thomas [3], Boolean models have been increasingly applied to biological signalling and regulatory networks [4–7]. With the rise of high-throughput functional genomic methods and the development of molecular pathway knowledge databases, modellers are now coping with networks encompassing hundreds of regulatory components controlling cell fate decisions for normal and pathological conditions (e.g. [8]).

In parallel, dozens of computational tools have been developed to infer, edit and analyse the dynamical properties of Boolean networks, using different programming languages and model formats (e.g. [9] and references therein). However, like experimental biologists, modellers are facing serious reproducibility challenges, which have been stressed in recent years (see [10], focusing on modelling studies using ordinary differential equations).

About a decade ago, several international teams working on the development of Boolean modelling tools gathered efforts to address these challenges. A first effort focused on the definition of a common standard format to foster the exchange and reuse of qualitative models between different software tools, which took the form of a dedicated extension of a new, modular release of the popular *SBML* format, known as *sbml-qual* [11].

**Figure 1.**
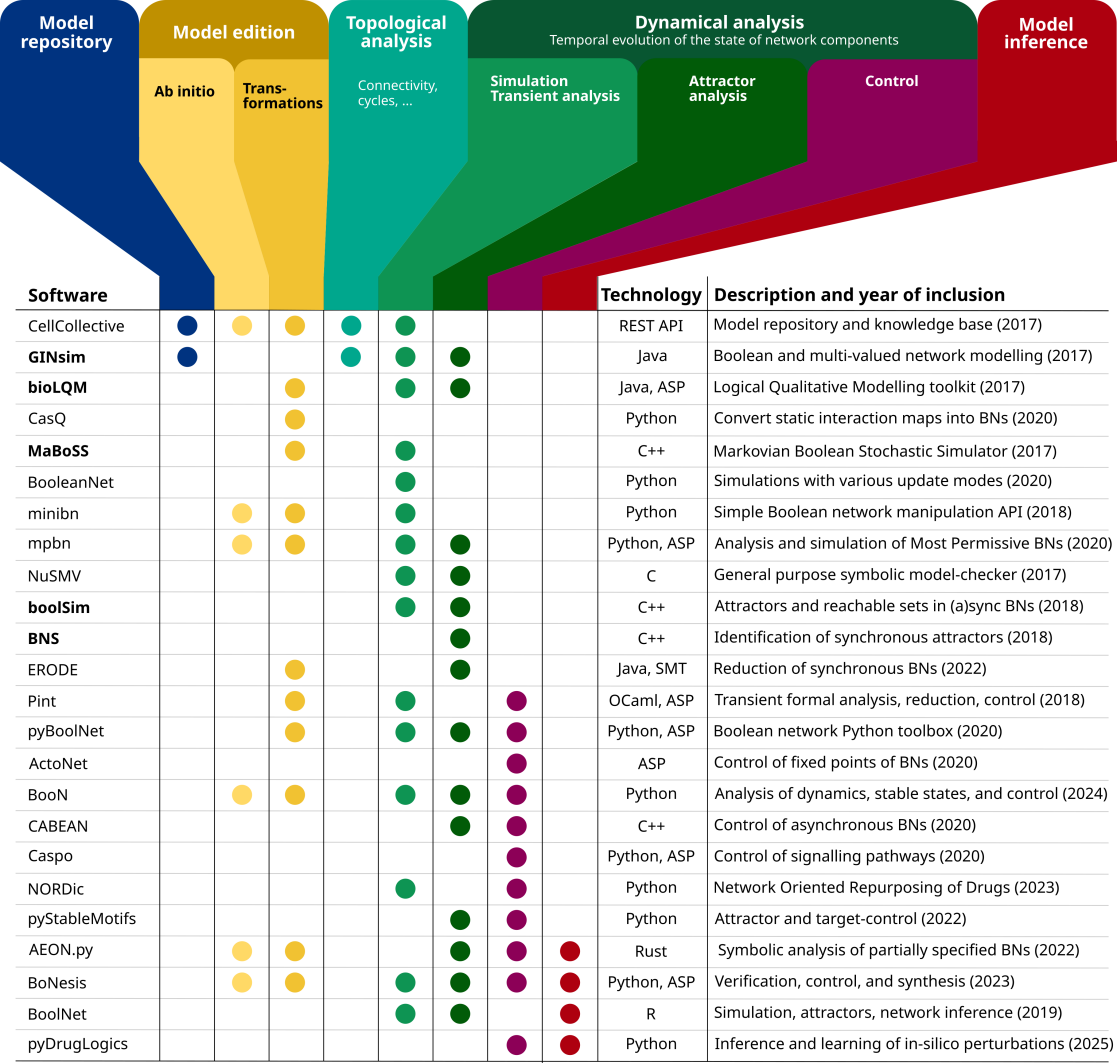
Tools currently integrated in the *CoLoMoTo* suite. Names in bold refer to the *CoLoMoTo* tools used in this tutorial.

A second, recurrent effort is devoted to the integration of a growing set of tools in a *Docker* container to ease their installation and articulation in flexible analysis workflows. The first release of the resulting *Common Logical Modelling Toolbox* (or *CoLoMoTo*) encompassed six complementary software tools [12]. Since then, the number of tools integrated into the *CoLoMoTo* suite has been constantly increasing, reaching a total of 24 in 2025. The tools currently integrated in the suite are listed in figure 1, with key characteristics.

This tutorial aims to help modellers take advantage of the multiple tools integrated into the *CoLoMoTo* software suite and perform sophisticated Boolean model analyses of complex signalling/regulatory networks. This tutorial relies on a comprehensive model of key signalling pathways and a core regulatory network controlling mammalian cell proliferation, published by Sizek *et al*. [13]. The proposed tasks performed with selected tools aim at comparing the model outcomes with expected experimental behaviours (e.g. existence of cell states in response to receptor activation, cell differentiation, etc.), predicting the results of potential perturbations (such as mutations in patients) or anticipating the effect of treatments.

As we shall see, the tutorial includes chunks of *Python* code to reproduce several of the results and figures published in the original article, and further extends these results with the help of several tools included in the *CoLoMoTo* software suite, in particular taking advantage of the *MaBoSS* package to perform stochastic simulations for the wild-type (WT) and various mutant backgrounds. This tutorial specifically aims to demonstrate the added value of combining different tools to perform reproducible dynamic analyses, and shows the effectiveness of using *Python* to orchestrate these different analyses.

## 2. Materials and methods

### 2.1. Installation

The notebook can be run on a recent personal computer with at least 16 Go of RAM and about 10 Go of available disk space.

The *CoLoMoTo* suite can be installed either as a *Docker* container or as individual *conda* packages.

#### 2.1.1. Installation of the *CoLoMoTo Docker* container

*Docker* allows packaging a complete pre-installed software environment, so that applications can run consistently across different machines and operating systems. As such, it is well adapted to reproduce complex computational workflows, relying on numerous software.

*Docker* and *Python* need to be pre-installed. Go to your folder and then type in a terminal:

**Figure:**
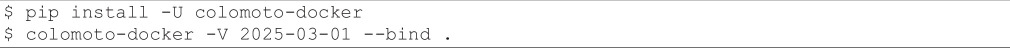


where $ stands for the terminal prompt.

#### 2.1.2. Installation of *CoLoMoTo conda* packages

As an alternative to using *Docker*, one can rely on *conda* to create a software environment able to reproduce the notebook on *Linux* and *macOS* computers by typing the following commands in a terminal:

**Figure:**
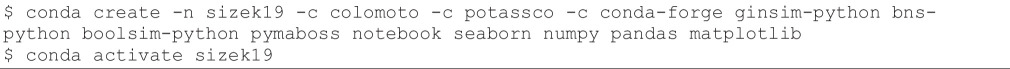


To launch this notebook, type the command:

**Figure:**
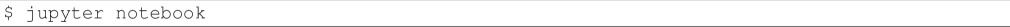


Because *BNS* and *boolSim* executables are not available for *Windows^®^* operating systems, the complete notebook can be executed only within a *Docker* environment on these systems.

More generally, as the versions of package dependencies cannot be fully controlled with the aforementioned *conda* command, reproducibility is better ensured by using the *Docker* container.

### 2.2. Model

The model published by Sizek *et al.* [13] has been imported and edited with the *GINsim* software [14]. It is publicly available in the GINsim *zginml* model format (including the layout, to be open with *GINsim*, version ≥ 3.0), as well as in the *sbml-qual* format, in the *GINsim* model repository (http://ginsim.org/). Hence, the model can be directly loaded from the notebook using the link to the corresponding *GINsim* model repository entry. Alternatively, the model can be downloaded locally and the loading command modified accordingly, thereby enabling offline work.

**Figure 2.**
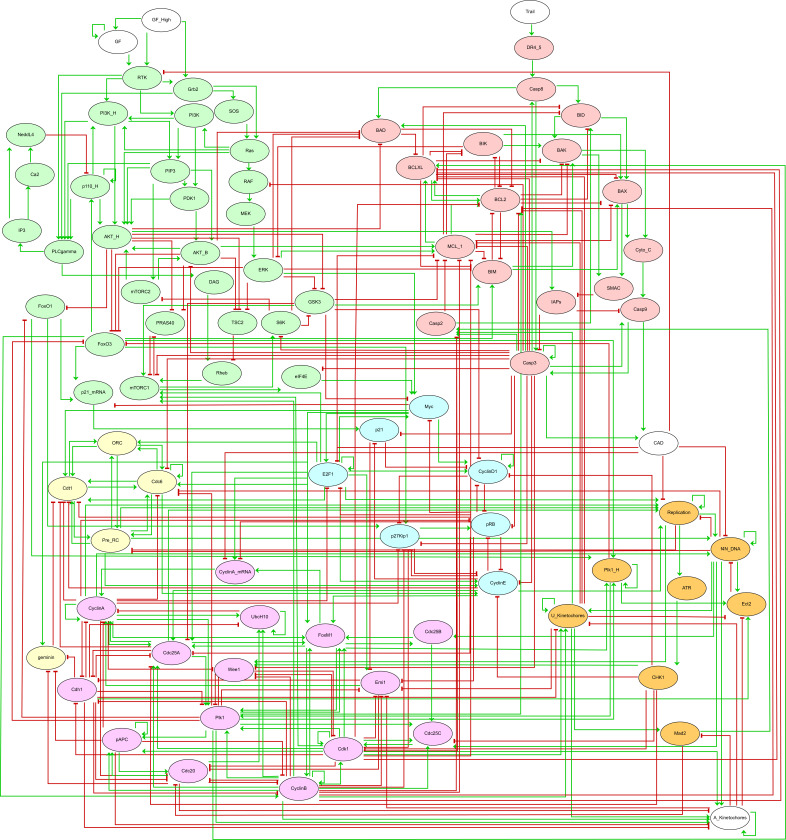
Boolean model published by Sizek *et al*. [13]. The corresponding regulatory graph encompasses 87 nodes, representing different molecular species and cellular processes involved in the regulation of cell proliferation and apoptosis in a generic human cell. Green: growth signalling module; red: apoptosis module; yellow: origin of replication licensing module; blue: restriction point module; pink: cell phase control module; orange: nodes connecting different modules/processes; white: environmental factors. Green arrows and red blunt arcs denote activatory and inhibitory interactions, respectively.

The model was initially conceived as the combination of five main signalling/regulatory modules contributing to the decision between cell proliferation versus death. A first module accounts for the orchestration of the cell cycle, resulting in the successive activation of the main markers of the cell division process, the cyclins. A second module accounts for the control of apoptosis, represented by the activation of Casp3 and the influence of the TRAIL death signal. A third module represents the regulation of growth via the MAPK and PI3k/AKT pathways and their activation by environmental factors. A fourth module represents the G1 restriction point. A fifth module represents the regulation of the origin of replication. Connecting all these modules (displayed with different colours in figure 2), the model can account for many different cellular situations, including genetic alterations common in cancer, and can successfully reproduce observed phenotypes.

## 3. Model analysis

The following sections of the tutorial cover the following steps:

— Loading of the packages required for the tutorial.
— Loading of the model and visualization of the network with the software *GINsim*.
— Attractor analysis with the software *bioLQM*.
— Computation of synchronous attractors with *BNS*.
— Analysis of the attractors of the model modules using *bioLQM*, *BNS* and *boolSim*.
— Stochastic dynamical simulations of the WT and mutant models with *MaBoSS*.
— The construction of compressed probabilistic transition graphs with *MaBoSS*.

This chaining of steps constitutes a coherent analysis workflow, starting with the identification of attractors (stable states or cycles), considering different updating schemes, before performing more precise and complex stochastic simulations, for the WT case, as well as for selected mutant backgrounds. Each step is performed using one selected software tool, but other *CoLoMoTo* tools could be used to perform similar analyses (cf. figure 1).

### 3.1. Loading required packages

Before running the analyses, the packages to be used need to be loaded. For this tutorial, only the necessary *CoLoMoTo* packages are selected. The other packages listed in figure 1 can be loaded according to the instructions provided at https://colomoto.github.io/colomoto-docker/.html. Note that these packages can also be loaded later on, just before their use.

Pieces of code will be shown in this tutorial in boxes as follows. They represent code cells extracted from the *Python Jupyter* notebook.

**Figure:**
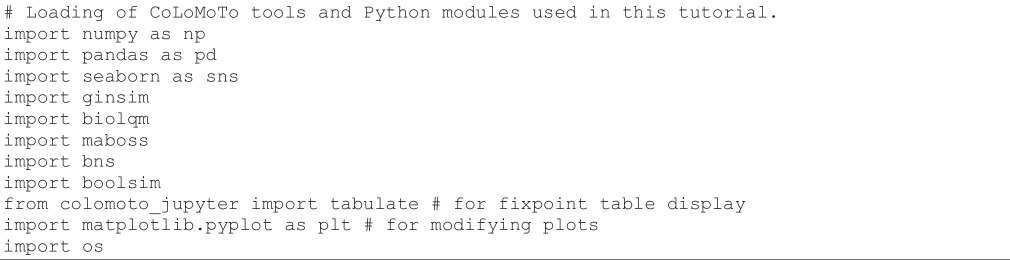


The execution of this cell outputs a few lines specifying what docker image and how many computer resources were used:

**Figure:**
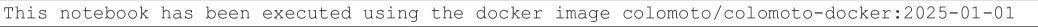


### 3.2. Loading and visualization of the model with *GINsim*

Prior to this tutorial, the model was first imported using *GINsim*, in order to redesign its layout and to associate extensive annotations with each regulatory node. The resulting model has the same logical rules as the original one and can be found in the *GINsim* repository at: http://ginsim.org/node/258.

To load the model and visualize its *regulatory graph*, we run the following code cell:

**Figure:**
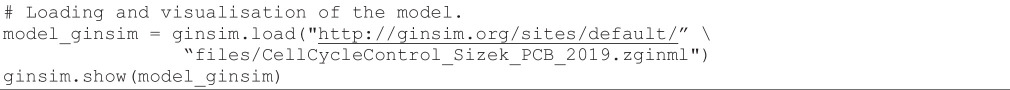


Figure 2 displays the corresponding regulatory graph.

The regulatory graph shown in figure 2 encompasses 87 nodes representing different molecular species and cellular processes involved in the regulation of cell proliferation and apoptosis in a generic human cell. The authors conceived this complex model as an association of five main functional modules:

— The growth signalling module (green nodes) incorporates growth signalling pathways driving cell cycle commitment, responsible for modelling the dynamics of PI3K, AKT1, MAPK and mTORC.
— The restriction switch module (blue nodes) is responsible for commitment to DNA synthesis.
— The origin licensing switch module (yellow nodes) controls licensing and firing of replication origins.
— The phase switch module (pink nodes) controls cell cycle progression (G1 → S → G2 → M), taking into account the role of Polo-like kinase 1 (Plk1) during mitosis.
— The apoptosis switch module (red nodes) implements the decision between cell survival versus apoptosis.

In the regulatory graph, green arrows denote activatory interactions, while red blunt arcs represent inhibitions. As many model nodes are regulated by multiple nodes, their responses to regulatory input node levels are encoded in Boolean rules, which combine literals (i.e. node ids) with the classical Boolean operators NOT (written ‘!’ in *GINsim*), OR (‘|’) and AND (‘&’). The rules defined in the initial publication were encoded in the *zginml* file available in the *GINsim* model repository.

### 3.3. Attractor analysis with *bioLQM*

*Attractors* represent the long-term (*asymptotic*) behaviours of the model. In the case of Boolean models, attractors can correspond to stable states (with each of the components frozen at level 0 or 1) or to periodic/cyclic behaviour (with a subset of components switching between values 0 or 1). Attractors are often associated with cellular phenotypes, cell fates (e.g. cell proliferation or cell death), or cellular types in the case of cell differentiation models. Their analysis allows the modeller to verify that the model reproduces the dynamical properties expected for the biological system under study, for normal or perturbed conditions.

To obtain a first overview of the asymptotic properties of the model, we can use the software tool *bioLQM* [15], which includes an efficient algorithm to identify all stable states [16]. The model is first converted into the *bioLQM* format, which can be achieved with the following code cell:

**Figure:**
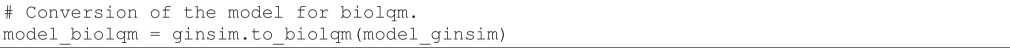


Using *bioLQM*, the *stable states* of the model can be computed by the following code cell:

**Figure:**
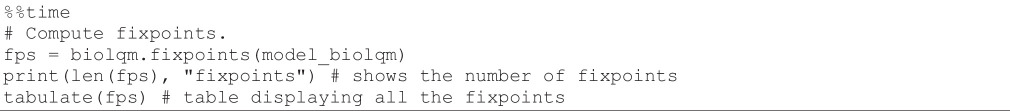


The execution of this code cell takes less than a second and outputs a table listing the eight stable states (also called *fixed points*) of the model (shown in the notebook), matching those reported by Sizek *et al*. in the supplementary table S2 of their article.

To better visualize any of these stable states, the corresponding values can be projected on the model regulatory graph with a simple code line, using *GINsim*:

**Figure:**
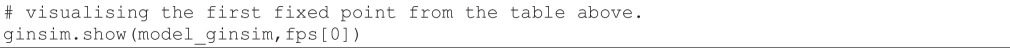


**Figure 3.**
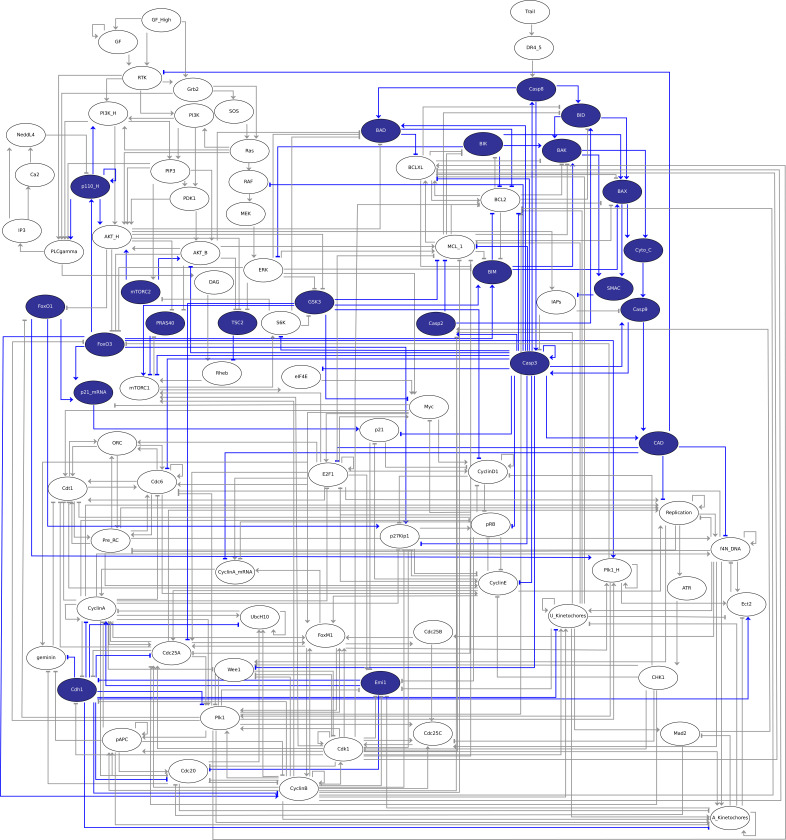
Graphical representation of a stable state of the Boolean model published by Sizek *et al*. [13] corresponding to a cell death phenotype. Active nodes and their outgoing edges are displayed in blue.

Such a visualization highlights the pathways/modules that are active in one particular stable state. An example of such stable state projection is provided in figure 3.

### 3.4. Computation of synchronous attractors with *BNS*

In their publication, Sizek *et al*. report the existence of a cyclic behaviour when a synchronous simulation method is used. Synchronous simulations simultaneously update all the components at a given state, according to the logical rules, leading to a unique successor state. This is a strong assumption, as in reality, timing plays an important role in biology and concurrent events barely occur simultaneously.

In contrast, asynchronous simulations typically update only one component at each time step, either selecting randomly one component to update, or yet considering separately all single update calls, potentially giving rise to more complex, non-deterministic dynamics.

Of note, for a given Boolean model, the stable states are not affected by the choice of updating, as stable states are devoid of update calls.

Cyclic attractors correspond to a circular sequence of events, such as the activation of cyclins, markers of the cell cycle. Cyclic attractors may depend on the choice of updating, and their identification is more complex in the asynchronous updating case.

*BNS* can be used to compute the synchronous attractors of the model (i.e. fixed points, as well as simple cyclic trajectories in the synchronous case). Although *BNS* can search for attractors of any length, this search fails with such a complex model. To overcome this limitation, a range of cycle lengths is defined, and attractors for the corresponding lengths are explored sequentially.

In the following code cell, we consider a maximum length of 32, and use *BNS* to compute all cycles of lengths between 1 to this maximum length. Of note, an attractor of length k is also an attractor of length 2k, 3k, etc. In particular, fixed points are detected as attractors of any length. Hence, in the following code cell, we further filter the attractors that amount to iterations of smaller ones.

**Figure:**
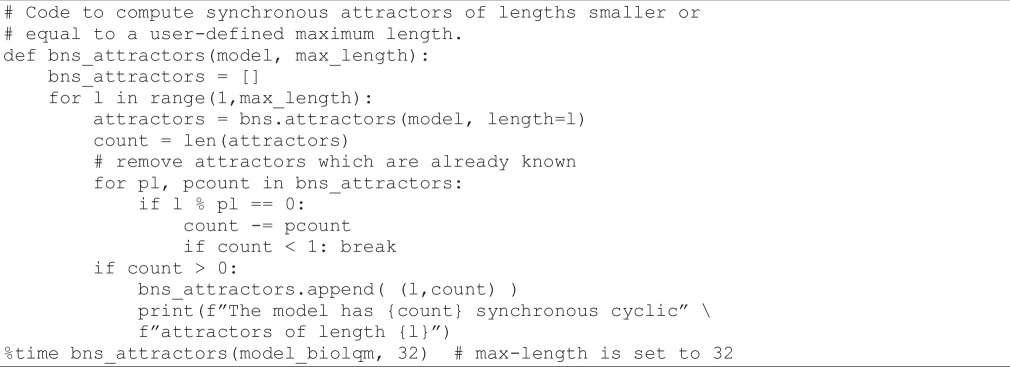


In addition to the eight fixed points already identified (corresponding to *cyclic attractors of length 1*), *BNS* returns four attractors of length 10, and one attractor of length 21, which correspond to the cyclic attractor reported by Sizek *et al*. in electronic supplementary material, table S2, supposedly representing the successive phases of the cell cycle: G1 from step 1 to 5, S for steps 6 and 7, G2 from step 8 to 13, prometaphase for step 14, metaphase from step 15 to 18, anaphase for step 19, telophase for step 20 and cytokinesis for step 21.

However, the interpretation of cyclic attractors under the synchronous updating strategy is not that straightforward. Indeed, the cyclic attractors of length 10 might be artefactual, as synchronous updating is known to give rise to spurious cycles.

In the following section, we further characterize the attractors of the five main constitutive regulatory modules, as well as the presence of coupling between these modules.

In §3.7, we will compute the probability of cyclic attractors under an asynchronous mode to follow the successive activations of the cyclins, each representing a phase of the cell cycle.

### 3.5. Analysis of the model constitutive modules

To generate their complex model, Sizek e*t al*. initially developed smaller models for different functional modules (cf. §2). These modules correspond to processes such as DNA damage, cell cycle, MAPK pathway or apoptosis and encompass the main genes that are known to be involved in these processes. They are defined by the authors in electronic supplementary material. The following code cell retrieves the information on the node composition of each module, counts the total number of nodes belonging to each of these modules, together with the number of nodes that belong to the rest of the network and that regulate nodes belonging to the module (also known as module *inputs*). It then computes the number of stable states, the number of terminal trap spaces (i.e. sub-hypercubes closed by the logical rules, comprising the stable states, but potentially also approximations to cyclic attractors), as well as the numbers of synchronous and asynchronous attractors for each of these modules.

**Figure:**
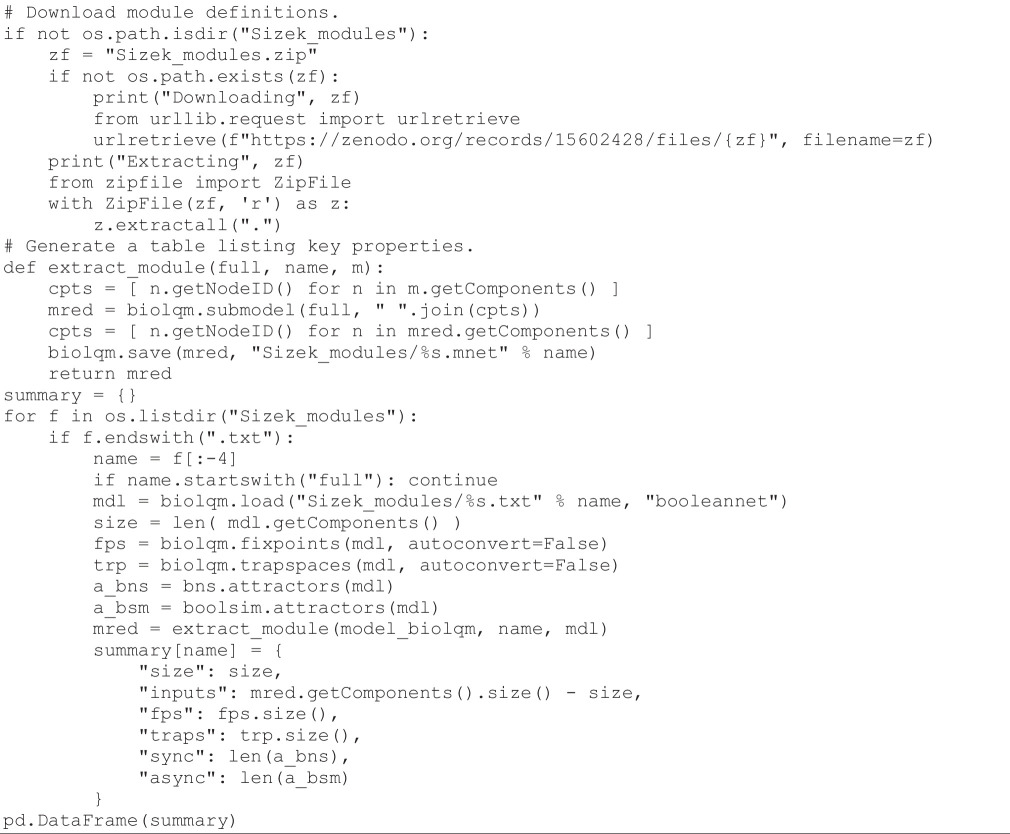


The execution of this code cell takes less than a second to generate the results reported in table 1.

**Table 1.** Some properties of the five modules constitutive of the model of Sizek *et al*. [13].

	restriction switch	apoptotic switch	PI3K	origin licensing switch	phase switch
number of nodes	7	16	8	4	15
number of input nodes	14	13	11	11	16
number of stable states	2	2	0	2	3
number of terminal traps	2	2	1	2	3
dynchronous attractors	2	2	1	2	3
asynchronous attractors	2	2	1	2	3

We can observe that the modules are highly interconnected, as they all have high numbers of inputs relative to their sizes, due to regulations exerted by nodes belonging to other modules.

Furthermore, all these modules but one display multistability, but no cyclic behaviour. The PI3K module is the exception, as it has a unique, cyclic attractor, regardless of the use of synchronous or asynchronous updating. The cyclic behaviour of the PI3K module is indeed reported and discussed by Sizek *et al.* [13].

In conclusion, the attractors found for the different modules do not help us understand the mechanisms underlying the cyclic behaviour of the whole model. More precisely, at this point, it becomes apparent that the global cyclic behaviour corresponding to the cell cycle (see §3.4) arises from the interconnections of multiple modules.

### 3.6. Characterization of synchronous cyclic properties with *bioLQM*

To interpret the meaning of a cyclic attractor, it is important to check which variables are fixed in this attractor and which ones switch values.

To visualize the 21 states composing the synchronous cyclic attractor, *bioLQM* can be used to generate a trace from one of the cycle states reported by Sizek *et al.* [13], considering a number of steps somewhat greater than 21. First, we define an initial state belonging to the cyclic attractor, according to Sizek *et al.* [13], with the following code cell:

**Figure:**
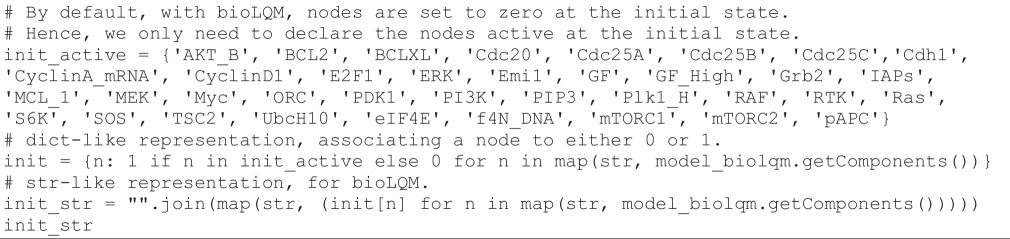


Next, we compute a synchronous updating trace, starting with this initial state and considering a maximum of 50 steps, with the following code cell:

**Figure:**
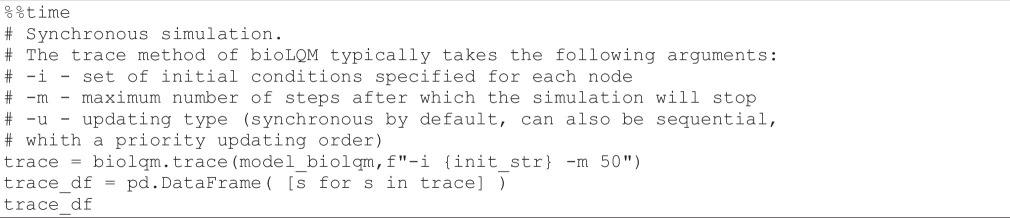


The execution of this cell takes less than a second and generates a table encompassing 22 rows (cf. notebook), each corresponding to one state belonging to a cyclic sequence. We can easily check that the first and last states are indeed identical with the following code cell:

**Figure:**
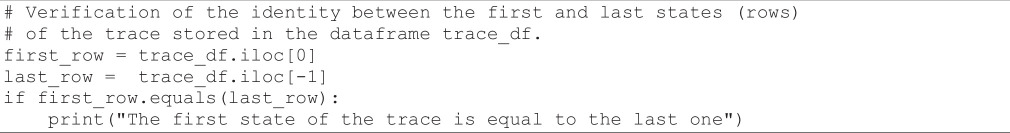


**Figure 4.**
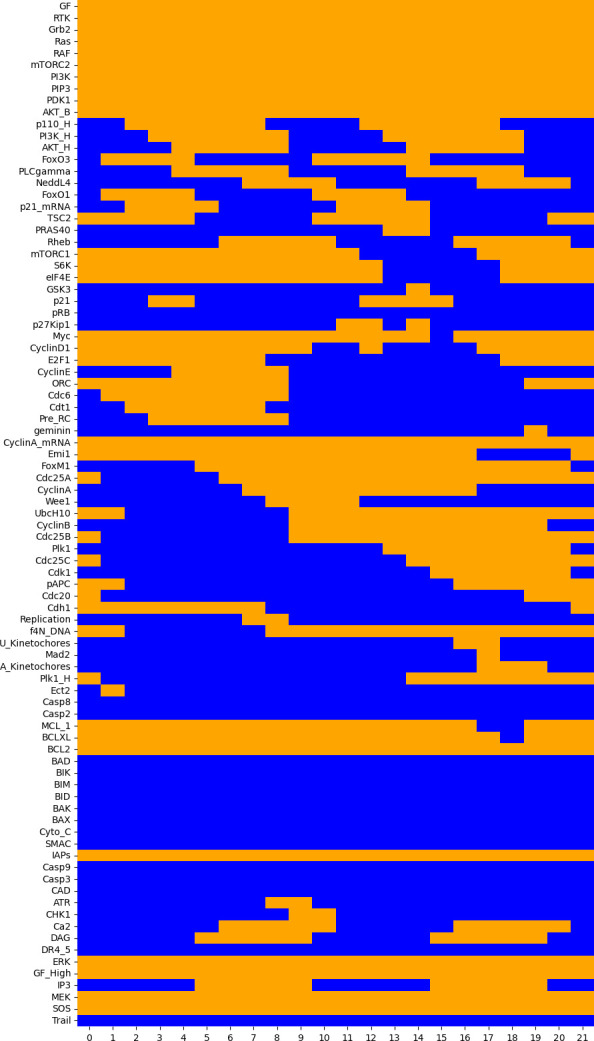
Heat map displaying the sequence of 22 states (columns) forming the synchronous cycle of length 21, with the initial state (first column, index 0) matching the last one (column with index 21). Orange cells denote the active components (rows) at the corresponding cycle state (column), whereas the blue cells denote the suppressed components.

The execution of this cell confirms that we have identified the cyclic attractor of length 21 mentioned above. To ease the comparison of this sequence of states with the periodic pattern reported by Sizek *et al.* [13] in fig. 4, we can plot it with similar graphical conventions, using the following code cell:

**Figure:**
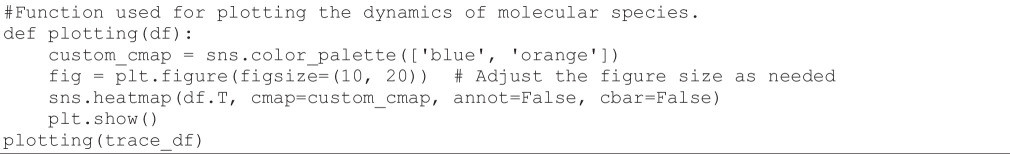


The resulting heatmap is shown in figure 4, exhibiting the full states sequence of the aforementioned cyclic attractor.

Note that some nodes are never activated along the cycle, while others are always activated. In particular, the apoptotic pathway (BIM, BAX, BAK, CytC, Casp3, Casp9, etc.) is inactive in this sequence, while the cell cycle is activated by the MAPK pathway (RTK, Ras, Raf, PI3K, etc.).

Hence, using different tools, we could also identify the synchronous attractor reported by Sizek *et al.* [13], which recapitulates the typical sequence of (in)activation of cell components, including cyclins. However, as this synchronous cyclic attractor is not expected to precisely reproduce the biological cyclic behaviour, a deeper analysis of the timing of cyclin (in)activations will be considered using asynchronous updating in §3.7.

Of note, Sizek *et al.* [13] reported that the observed cyclic behaviour is sensitive to the updating strategy used. To further characterize this sensitivity, *bioLQM* can be further used to compute traces starting from the same initial state but using random asynchronous updating, i.e. selecting one node among all nodes called to be updated at each state, with identical probabilities assigned to all single node transitions. This can be achieved using the following code cell:

**Figure:**
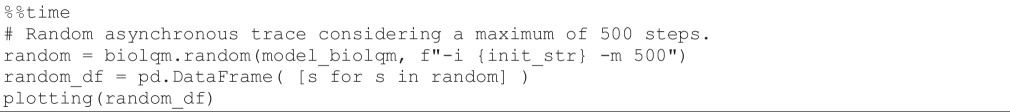


**Figure 5.**
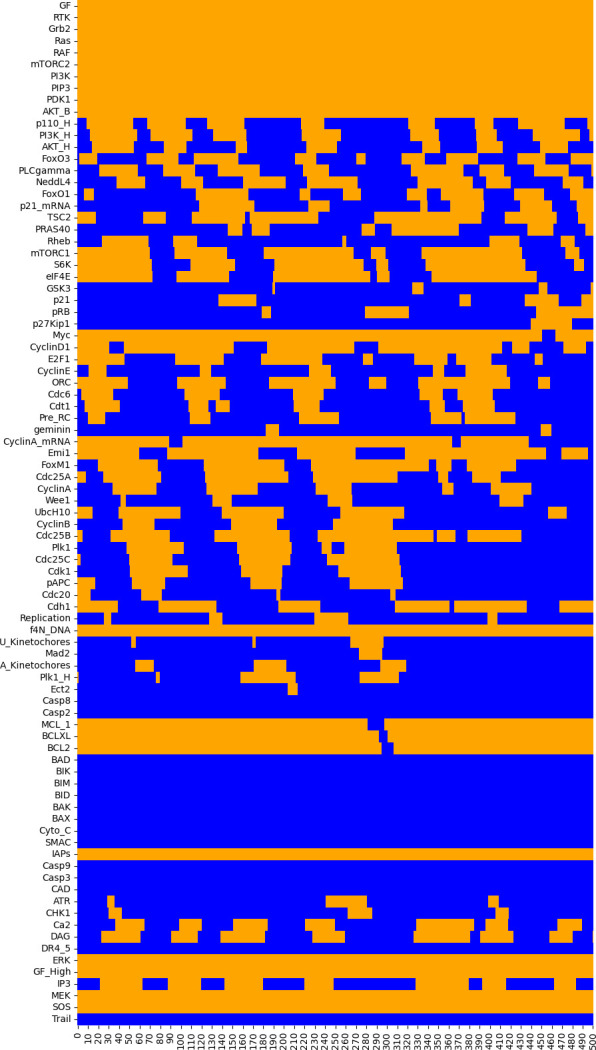
Heat map displaying a sequence of 500 states (columns) resulting from a random asynchronous updating of the model, starting with the same initial state shown in figure 4. Orange cells denote the active components (rows) at the corresponding cycle state (column), whereas the blue cells denote the suppressed components.

This cell takes a few seconds to execute. Note that a longer maximal number of steps (500) is considered here because we do not know if the trace will end up in an asynchronous cycle, or how long such a cycle might be. A typical result is shown in figure 5.

In the heatmap shown in figure 5, we can observe a transient oscillating pattern, but this pattern gets progressively degraded along the simulation. This is due to the random choice of transitions whenever several components are called to switch their values at a given state, according to their logical rules. Of note, if we repeat the same random asynchronous simulation, we typically end up with a different transient oscillating pattern.

Interestingly, when large enough maximal step numbers are considered, simulations ultimately lead to a stable state corresponding to an apoptotic fate. To visualize this, in the notebook, we provide the code for a simulation using a maximum number of steps set to 2000, which needs to be uncommented to be run.

In their paper, the authors defined a biased transition order to enforce a more robust cyclic behaviour. Hereafter, we propose an alternative approach to deepen our understanding of the asynchronous behaviour of the model, taking advantage of the software *MaBoSS* [17] to integrate over a large number of potentially different traces.

### 3.7. Using *MaBoSS* software to perform stochastic simulations of the wild-type model

It is difficult to build the asynchronous state transition graph and analyse the transient dynamics of such complex models. To cope with this issue, we use *MaBoSS*, a tool performing stochastic simulations over Boolean networks based on the continuous-time *Markov chain* formalism, relying on the *Gillespie algorithm* [17]. The software enables the generation of *time plots* showing the evolution of mean component values (or patterns thereof), as well as of *pie charts* showing the probabilities of the different model states reached at the end of the simulation.

The *bioLQM* model can be converted into the format required by *MaBoSS* with the following code cell:

**Figure:**
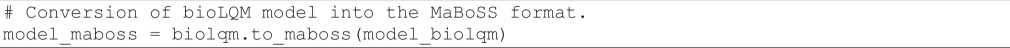


After loading the model, simulation parameters can be modified with the following code cell:

**Figure:**
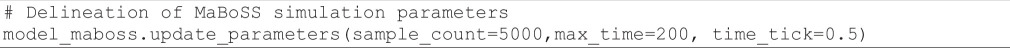


This code cell defines the following simulation parameters:

— Number of simulations: 5000.
— Maximum length of the simulation (in arbitrary units): 200.
— Interval between two time points: 0.5.

Note that *MaBoSS* uses continuous-time Boolean modelling and requires the specification of a maximum simulation length and an interval between time points. All the stochastic simulations reported in the notebook use these same simulation parameters.

Next, we define a WT version of the model, called *WT*, and we select the *external variables*, i.e. the variables for which the values will be reported during the simulation. This is crucial to avoid combinatorial explosion.

**Figure:**
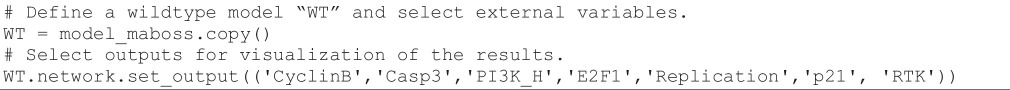


Note that when exporting a model to *MaBoSS* with *bioLQM* or *GINsim*, all the initial values are set to 0 by default. Hereafter, we assign to the model the initial conditions defined by Sizek *et al.* [13] in their fig. 6.

**Figure:**
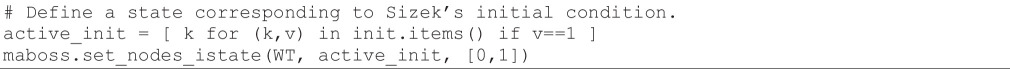


**Figure 6.**
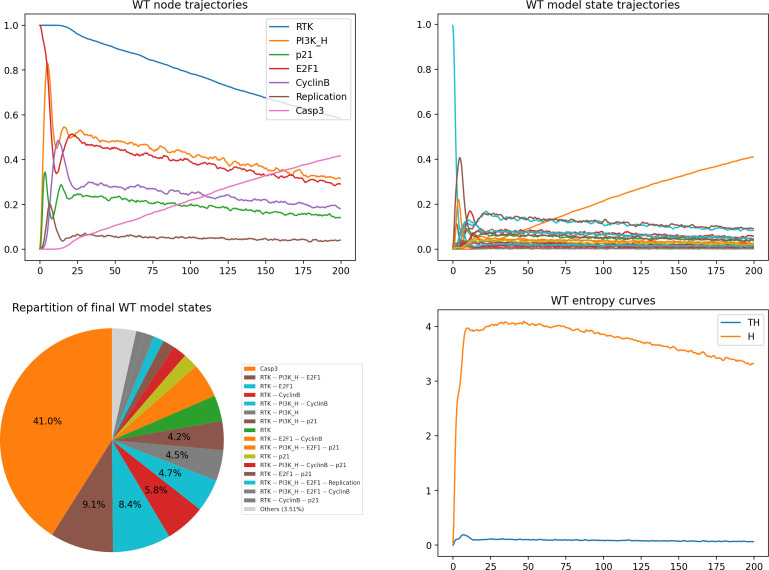
*MaBoSS* simulations of the WT model. Upper left panel: trajectories for a subset of nodes. Upper right panel: trajectories for a subset of model states (legend in the lower left panel). Lower left panel: pie chart representing the probabilities for the model states at maximum time (time = 200). Lower right panel: entropy (H) and transition entropy (TH) time plots.

*MaBoSS* simulation results can be graphically displayed as *time plots* showing the probabilities of active nodes or model states (vectors of active nodes) over time, or as *pie charts* showing the probabilities of the final states (i.e. the states reached at the end of the simulations). In brief, the trajectories plotted on these graphs represent mean trajectories of 5000 single trajectories focusing on a subset of seven selected nodes. It is possible to visualize the activity of each of these nodes over time (figure 6, upper left), or the evolution of model states defined as configurations of active nodes (figure 6, upper right), which can also be displayed as a pie chart for the last time point considered (figure 6, lower left).

It is also possible to compute the *state entropy* (H) and the *transition entropy* (TH) over time (figure 6, lower right), which can be interpreted as signatures for the stable states or cyclic attractors. These curves provide a means to check the nature of the attractors of the model. Entropy measures the ‘disorder’ of the system studied. A very low transition entropy at the end of a simulation thus suggests that the system has reached an equilibrium, while a positive state entropy may suggest the existence of a cyclic attractor or the occurrence of multiple stable states (for more details, see [18]).

The following code cell launches a simulation of the WT model with *MaBoSS*, using the parameters defined above:

**Figure:**
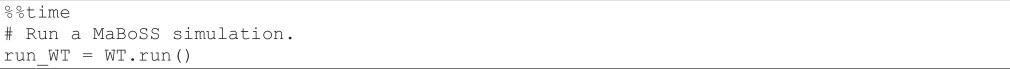


The execution of this code cell takes less than a minute. To graphically visualize the results of this simulation in terms of node or model states trajectories, of final model states repartition, and of entropy curves, we can use the following code cell:

**Figure:**
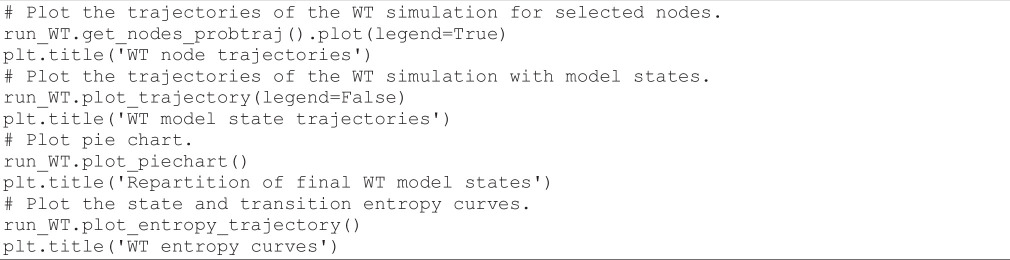


The resulting graphs are shown in figure 6.

The simulations reveal a transient cyclic-like behaviour, which is eventually lost due to the spontaneous Casp3 activation, thereby revealing the progressive dominance of the apoptotic stable state.

The state entropy initially rises abruptly, and later slowly decreases. In parallel, the transition entropy curve displays a little peak before remaining close to zero, with some noise. Together, these two curves are suggestive of a transient periodic behaviour, which ultimately collapses as more and more cells trigger apoptosis.

Next, we can use *MaBoSS* to study the impact of changes in the initial conditions on the dynamic behaviour of the model. In the preceding simulation, all death signals were initially OFF. Hereafter, we assess the effect of activating TRAIL, a ligand binding to death receptors of tumour cells and initiating the death pathways, while keeping the rest of the other initial conditions unchanged. Note that TRAIL is an input of the death module and thus not regulated. Such inputs are set to either 0 or 1 in the initial conditions, thereby conveying the status of the environment. The following code cell defines a copy of the WT model, triggers TRAIL level to 1 at the initial state, performs the simulation and finally displays the results:

**Figure:**
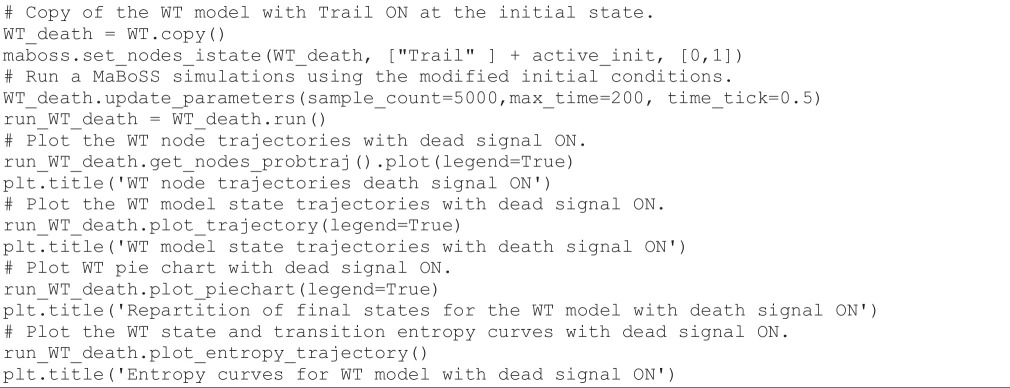


**Figure 7.**
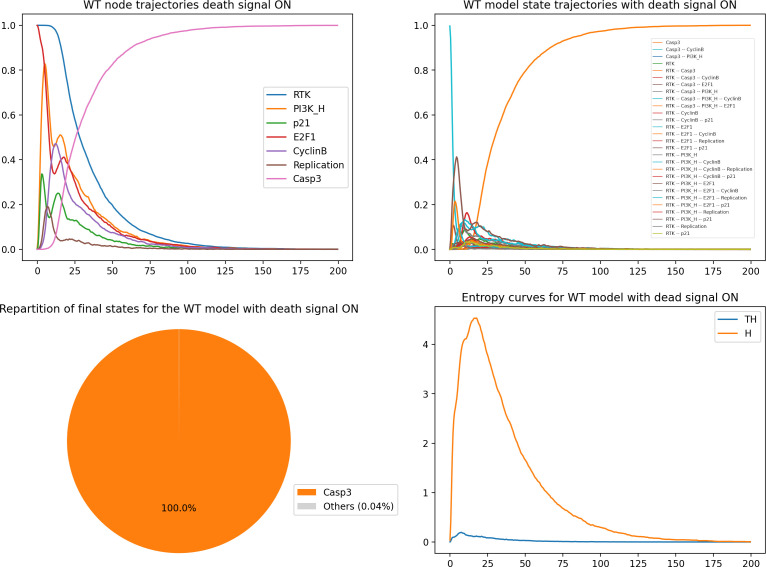
*MaBoSS* simulations for the WT condition with death receptor signals active. Upper left panel: trajectories for a subset of nodes. Upper right panel: trajectories for a subset of model states. Lower left panel: pie chart displaying the probabilities for the model states at maximum time (time = 200). Lower right panel: entropy (H) and transition entropy (TH) time plots.

The resulting graphs are displayed in figure 7.

Looking at the graphs of figure 7, we can see that the activation of TRAIL at the initial state enables a faster rise of Casp3 and thus of the apoptotic fate. In parallel, the state entropy drops much faster, while the transition entropy stabilizes around zero, suggesting a stable state situation.

### 3.8. Mutant simulations with *MaBoSS*

In their article, Sizek *et al.* report a sophisticated analysis of the role and dynamical properties of the PI3K/AKT1 pathway, and recapitulate deleterious effects of alterations thereof. They further analyse the impact of the blocking of the Polo-like kinase 1 (Plk1, a mitotic driver and chemotherapy target) on cell cycle progression.

Hereafter, we focus on the analysis of the impact of the perturbations of other model components, using the *MaBoSS* software. Such perturbations can be understood as mutations that patients bear, or as targets of drugs that would suppress proliferation or increase apoptosis.

#### 3.8.1. Stochastic simulation of ectopic Casp8 activity

Casp8 is a caspase initiating the apoptotic cascade. By changing its activity (ectopic activity or knockdown), we can explore its role in triggering cell death. Using the following code cell, we first simulate the impact of a constant activation of Casp8 and display the resulting time plot and pie chart.

**Figure:**
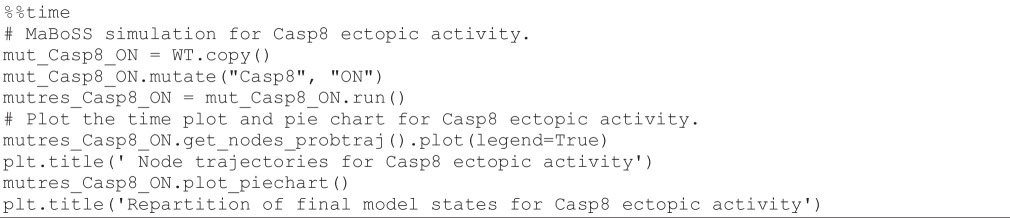


**Figure 8.**
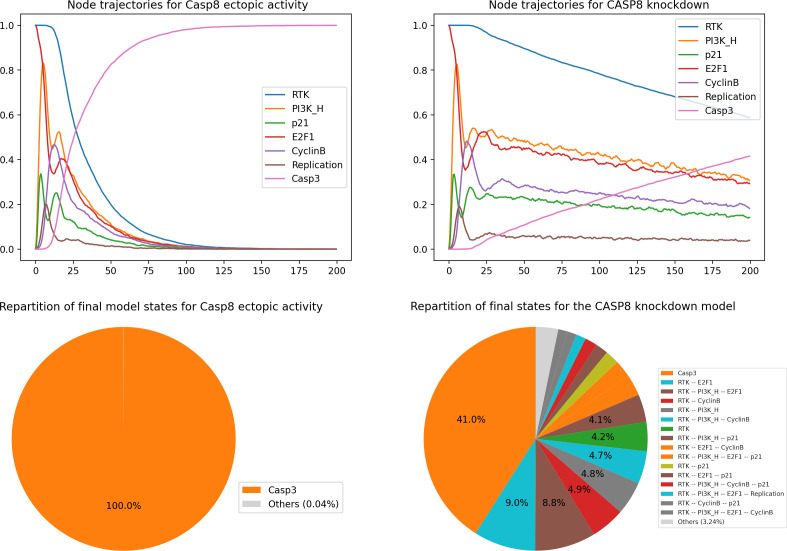
*MaBoSS* simulations for Casp8 mutants. Left panels: node trajectories and pie chart showing the asymptotic solutions for Casp8 overexpression. Right panels: node trajectories and pie chart representing the probabilities for the model states at the end of simulations for Casp8 knockdown.

Figure 8 (left) shows the resulting plots. Based on these results, we can conclude that the ectopic expression of Casp8 drives the cells into apoptosis faster, whether the death signal (TRAIL) is ON or OFF. This shows that death is not dependent on external signals in this context: the cell becomes independent from the death receptor engagement and apoptosis is constantly activated.

#### 3.8.2. Stochastic simulation of Casp8 knockdown

We can also check the impact of a full knockdown of Casp8 by executing the following code cell:

**Figure:**
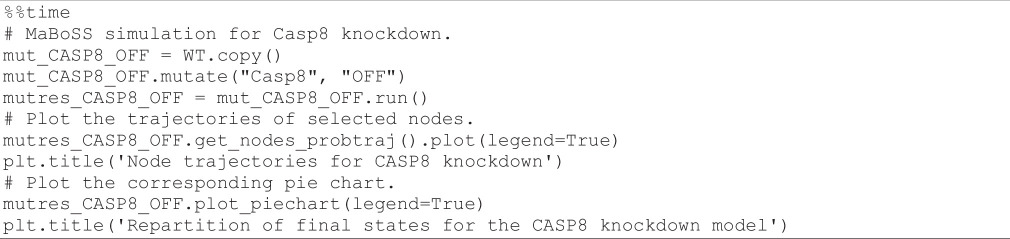


As shown in figure 8 (right), Casp3 is initially blocked but becomes ultimately activated, like in the WT case, independently of the status of Casp8. Hence, the model predicts that apoptosis (represented by Casp3 activity) can ultimately occur even in the absence of Casp8 activity. The knockout of Casp8 does not affect the model in the absence or presence of TRAIL.

Of note, it is possible to knock out a specific interaction with *bioLQM*, using the syntax: ‘target:regulator%0’, denoting a blockade of the interaction exerted by the regulator onto the target:

**Figure:**
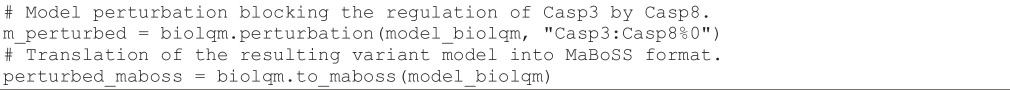


Using a bit of *Python* code, it is further possible to systematically explore a series of mutants, and to filter the results. In this respect, the notebook provides an example of such code, considering 10 mutant simulations (inhibition and activation of five different nodes), and filtering those enabling a blockade of apoptosis, denoted by the suppression of Casp3 activation, while keeping the cell cycle active. This would correspond to a highly proliferative phenotype, with some alterations correlating with cancer patient conditions.

Overall, this analysis shows the important but not necessary role of Casp8 in triggering apoptosis in this model. The gradual activation of Casp3, even in the absence of Casp8, may be an artefact of the model, or could be interpreted as an intrinsic propensity of cells to die after multiple rounds of divisions. In the latter case, the model would benefit from the inclusion of more details regarding the mechanisms triggering cell death.

### 3.9. Deeper analysis of the quasi-cyclic synchronous attractor

In this section, we explore the dynamics of the cell cycle by studying the succession of cyclin activations and inactivations. In continuous frameworks, i.e. models based on ordinary differential equations, it is possible to follow the activations of the cyclins that are crucial to ensure a proper cell cycle. In the WT situation, we expect that the cyclins get activated in the following order: CyclinE, followed by CyclinA, then CyclinB, and get inactivated in the same order. This sequence is not based on a simple cascade, but on complex regulation where new phases are controlled by checkpoints.

In the case of stochastic Boolean simulations, the verification of this sequence of cyclin activations is more challenging. In this respect, we have developed a functionality reporting the probabilities of occurrences of specific node level transitions, thereby enabling the delineation of highly probable orders of activations and inactivations of cyclins over time.

#### 3.9.1. Verification of the order of activations of cyclins in the wild-type situation by plotting a compressed state transition graph

The following code cell defines a simulation of the WT model focusing on cyclin dynamics:

**Figure:**
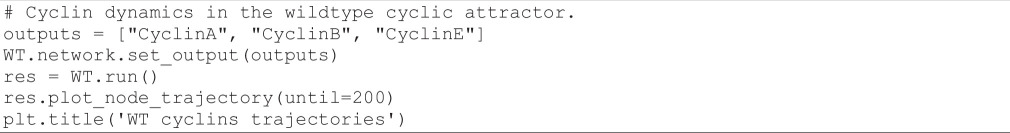


**Figure 9.**
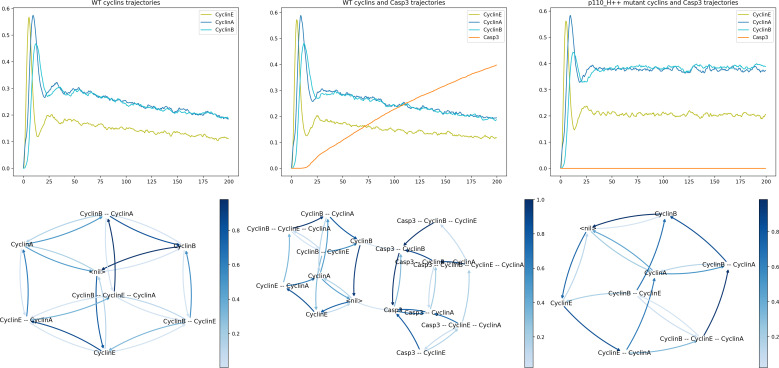
Left: *MaBoSS* simulation of the WT condition, showing the mean probabilities of cyclin activations and inactivations (upper left panel), along with a compressed state transition graph (lower left panel). Middle: *MaBoSS* simulation of the WT condition, showing the mean probabilities of cyclin and Casp3 activations and inactivations (upper middle panel), along with a compressed state transition graph (lower middle panel). Right: *MaBoSS* simulation of the p110++mutant, showing the mean probabilities of cyclin and Casp3 activations and inactivations (upper right panel), along with a compressed state transition graph (lower right panel). In the compressed state transition graphs, each node represents a set of states characterized by a specific combination of activated markers (i.e. cyclins A, B and E, plus Casp3 in the middle lower panel), whereas the colours of the edges denote the probabilities of transitions between pairs of sets of states (light blue: low probability; dark blue: high probability).

The resulting time plot is shown in figure 9 (top left). We can see that the cyclins follow the expected order with the proper sequence of peaks: first CyclinE around *t* = 5, then CyclinA around *t* = 10, and finally CyclinB around *t* = 15. Note that as *MaBoSS* reports mean probabilities over time, these oscillations are rapidly damped.

We can further compute the frequencies of cyclin activations and inactivations over all individual trajectories of the simulation. To do this, we use a new functionality of *MaBoSS*, estimating the frequencies of transitions between *activity patterns* involving selected nodes, and plot them as a graph. To prevent the explosion of the size of this graph, we only use the three main cyclins responsible for the cyclic attractor. The following code cell computes such transition frequencies and displays the results as a *compressed state transition graph*, i.e. a projection of the transition frequencies computed over the full state space onto the cyclin activity patterns of interest.

**Figure:**
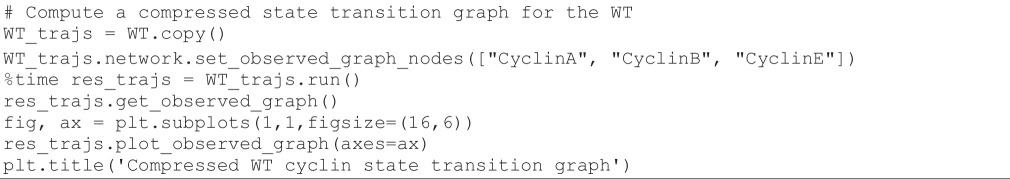


The resulting transition graph is shown in figure 9 (bottom left).

In this graph, the transitions denoted by darker blue arrows have a higher probability of occurring. This representation enables us to visualize the most probable sequence of cyclin activations and inactivations. Starting from the state ‘<nil>’ (corresponding to G0), the most probable sequence of state transitions successively involves CyclinE activation, CyclinA activation, CyclinE inactivation, CyclinB activation, CyclinA inactivation and finally CyclinB inactivation, thereby completing the cycle. Note that other cycles can be followed with lower probabilities.

#### 3.9.2. Analysis of the apoptosis triggering timing along the cell cycle

Here, we study the condition leading to apoptotic death, focusing on the point in the cell cycle where Casp3 is activated. We can achieve this using the following code cell:

**Figure:**
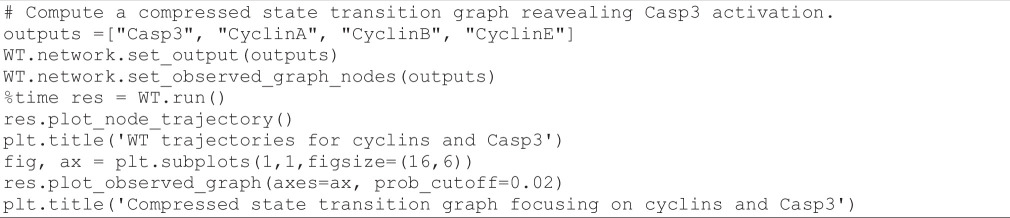


The resulting graphs are shown in figure 9 (middle). In the time plot (figure 9, middle top), we can observe that cells initially follow the expected cycle, but quickly activate Casp3 and are thus led to apoptosis.

Turning to the compressed transition graph (figure 9, middle bottom), we used a minimum probability threshold (2%) to discard unlikely edges during plotting. We see that there are two main ways to activate apoptosis: from the node ‘<nil>’ (with no active cyclins), which presumably represents the G0 state, and from the node ‘cyclinB’ (with only cyclin B active), which tentatively represents the G2/M phase.

#### 3.9.3. Analysis of cyclin triggering timing in presence of ectopic p110_H activity

In their article, Sizek *et al.* [13] reported that p110 activates both PI3K and AKT, which in turn inhibits cell death. In the mutant analyses performed by these authors, p110 played a key role in the interaction between the MAPK module and the apoptotic module. To verify the impact of a forced p110_H activation, we can execute the following code cell:

**Figure:**
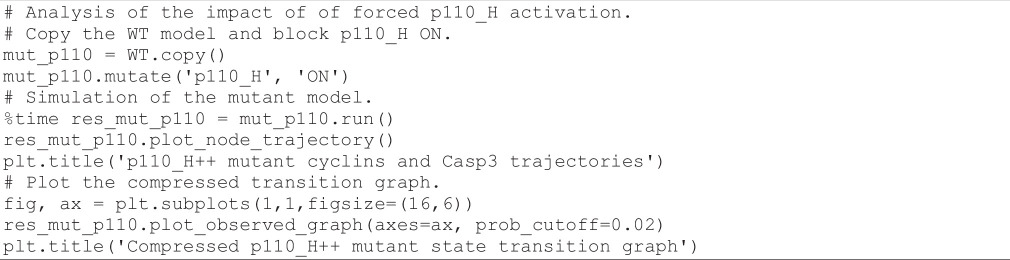


As can be seen in the resulting graph (figure 9, right), the ectopic activity of p110_H completely disables the activation of apoptosis, enabling the maintenance of the oscillatory behaviour.

## 4. Conclusions

The *CoLoMoTo* software suite integrates over 20 tools written in multiple languages, and makes them accessible with a single popular language (*Python*). After providing the instructions to install the suite, this tutorial focused on the analysis of a published model of the regulatory network controlling mammalian cell proliferation [13], which can be further used as a template for the analysis of any other Boolean model encoded in one of the multiple supported formats (e.g. *sbml-qual*).

The tutorial includes *Python* code enabling the reproduction of several of the results reported by the initial publication [13], as well as to further extend these results with new stochastic simulation results for WT cells, as well as for various single and double mutants. Using selected tools included in the *CoLoMoTo* suite, we showed how to compute the model attractors with synchronous or asynchronous updating. Although stable states are consistently preserved, our analysis emphasizes the sensitivity of synchronous cyclic attractors to the update mode.

We further showed how the use of a probabilistic simulation framework, implemented in the *MaBoSS* software, can provide interesting information on the transient behaviour of such a complex model, for WT conditions, as well as for different types of model perturbations.

Integrating all these analyses into an executable *Jupyter Notebook* greatly facilitates their reproducibility, as well as extensions. The notebook can also be used as a template for encoding completely new model analyses.

Due to space constraints, this notebook explicitly covers the use of only a fraction of the tools available in the *CoLoMoTo* suite. However, all integrated tools are accompanied by short specific tutorials available in the *CoLoMoTo Docker* container, as well as on the *CoLoMoTo* website (https://colomoto.github.io/). The analysis presented in this tutorial can be easily modified or extended by taking advantage of other tools included in the *CoLoMoTo* suite. For example, simulations with different updating modes can also be performed with *mpbn* or *BooleanNet*; *BooN* can be used for stable state analysis; *Pint* can be used to perform automatic search of mutations enforcing or impeding the reachability of a specific attractor; models can be imported from other databases, such as *Cell Collective*, in different formats, such as *BoolSim* or *BoolNet* formats.

## Data Availability

The notebook and companion files are available at Zenodo [19]. The source notebook file (with an ipynb extension) can be uploaded and executed within the Jupyter interface of the CoLoMoTo notebook, using the Docker image colomoto/colomoto-docker:2025-03-01. Supplementary material is available online [20].
